# Use of High-Dose Sertraline for Obsessive-Compulsive Disorder: A Case Report

**DOI:** 10.1192/j.eurpsy.2025.2074

**Published:** 2025-08-26

**Authors:** R. Fernández-Fernández, Á. Izquierdo de la Puente, P. del Sol Calderón, A. Rodríguez-Rodríguez, D. Gómez-Olmeda

**Affiliations:** 1 Hospital Universitario Infanta Cristina, Parla; 2 Hospital Universitario Puerta de Hierro Majadahonda, Majadahonda; 3 HM Puerta del Sur, Móstoles, Spain

## Abstract

**Introduction:**

The administration of high-dose sertraline for the treatment of obsessive-compulsive disorder has been investigated as a potentially more efficacious strategy in cases of treatment resistance, compared to standard dosing regimen. Studies have also evaluated the safety and tolerability of doses as high as 650 mg/day (Levy et al. Compr Psychiatry 2024; 133:152486).

**Objectives:**

To highlight the importance of understanding the potential use of high-dose sertraline for the treatment of treatment-resistant obsessive-compulsive disorder.

**Methods:**

Case report and literature review

**Results:**

This is a 50-year-old woman referred from the emergency department, where she presented with thoughts of death and was diagnosed with ‘Depressive Disorder. Impulsion Phobia without warning signs’ and treated with Sertraline 100 mg. During the first consultation, the patient reported the following symptoms:

- Marked emotional overwhelm and hypervigilance.
- Intrusive thoughts related to her father, generating significant guilt.
- Thoughts in which she feels the desire to harm herself.
- Compulsive need to check everything she writes, regardless of the context, to confirm she has not written anything bad about herself or her family.
- Compulsive need to review past actions to ensure she did not say anything inappropriate (despite not having spoken).
- Compulsive need to revisit conversations to ensure she had not said anything inappropriate, which eventually led her to stop speaking altogether.
- Mixed insomnia due to anticipatory anxiety about the thoughts she might have during the night.

During this initial visit, a diagnosis of Obsessive-Compulsive Disorder with mixed obsessive thoughts and acts (ICD-10; F42.2) was made. The Y-BOCS was administered, with the patient scoring 19 for obsessions and 12 for compulsions (total score: 31, indicating severe OCD). The dose of sertraline was increased to 300 mg, and aripiprazole was added. However, aripiprazole had to be discontinued after 15 days due to poor tolerance, and risperidone was introduced, which also had to be discontinued after 15 days due to poor tolerance.

After four months of follow-up and monotherapy with sertraline, the patient presented with almost complete resolution of symptoms, a stable mood, calm demeanor, and regained control over her thoughts, along with the disappearance of the compulsive checking behavior. A Y-BOCS was administered again, with a score of 8 for obsessions and 5 for compulsions (mild severity).

**Conclusions:**

The use of high-dose sertraline (250-400 mg/day) may be an effective alternative for maintaining monotherapy in patients with treatment-resistant obsessive-compulsive disorder. However, it is important to consider that previous studies have shown a better response but not a higher overall response rate (Ninan et al. J Clin Psychiatry 2006; 67:15-22).

**Disclosure of Interest:**

None Declared

